# Status of islet transplantation and innovations to sustainable outcomes: novel sites, cell sources, and drug delivery strategies

**DOI:** 10.3389/frtra.2024.1485444

**Published:** 2024-11-01

**Authors:** Jordan M. Wong, Andrew R. Pepper

**Affiliations:** Department of Surgery, Faculty of Medicine and Dentistry, University of Alberta, Edmonton, AB, Canada

**Keywords:** islet transplantation, diabetes, biomaterials, alternative site, immunosuppression

## Abstract

Islet transplantation (ITx) is an effective means to restore physiologic glycemic regulation in those living with type 1 diabetes; however, there are a handful of barriers that prevent the broad application of this functionally curative procedure. The restricted cell supply, requisite for life-long toxic immunosuppression, and significant immediate and gradual graft attrition limits the procedure to only those living with brittle diabetes. While intraportal ITx is the primary clinical site, portal vein-specific factors including low oxygen tension and the instant blood-mediated inflammatory reaction are detrimental to initial engraftment and long-term function. These factors among others prevent the procedure from granting recipients long-term insulin independence. Herein, we provide an overview of the status and limitations of ITx, and novel innovations that address the shortcomings presented. Despite the marked progress highlighted in the review from as early as the initial islet tissue transplantation in 1893, ongoing efforts to improve the procedure efficacy and success are also explored. Progress in identifying unlimited cell sources, more favourable transplant sites, and novel drug delivery strategies all work to broaden ITx application and reduce adverse outcomes. Exploring combination of these approaches may uncover synergies that can further advance the field of ITx in providing sustainable functional cures. Finally, the potential of biomaterial strategies to facilitate immune evasion and local immune modulation are featured and may underpin successful application in alternative transplant sites.

## 1 Introduction

The upward trend in diabetes mellitus has been a major worldwide health concern. With a 129.7% increase in the global prevalence of diabetes from 1990 to 2017, healthcare costs and disease morbidity are also on the rise (1). As of 2017, approximately ∼30% of Canadians are living with diabetes or prediabetes, and between 2011 and 2022 these cases are estimated to result in >$17 billion per year in associated healthcare costs (2). These striking statistics reflect the significant societal burden of diabetes, necessitating the development of treatments and solutions that reduce disease morbidity. Further, it has warranted thorough investigations into the pathophysiology behind this metabolic disease.

Diabetes mellitus is characterized as a metabolic disease with the central symptom of chronic hyperglycemia. Hyperglycemia is often a result of either decreased insulin secretion from the pancreas, defects in the body's response to insulin, or a combination of the two (3). As a result, chronic uncontrolled hyperglycemia can lead to long-term irreversible damage including both microvascular (retinopathy, nephropathy, and neuropathy, etc.) and macrovascular complications (coronary artery disease, cerebrovascular disease, peripheral vascular disease, etc.) (4). These late-stage diabetes complications, in addition to the strenuous diabetes therapies, have a significant negative impact on patients’ perceived quality-of-life (5). People suffering from chronic hyperglycemia can be categorized into two broad categories: type 1 diabetes mellitus (T1DM) which is manifested by the autoimmune destruction of insulin-secreting cells (pancreatic beta-cells), and type 2 diabetes mellitus (T2DM) which is a consequence of beta-cell dysfunction and the development of insulin resistance, with the latter accounting for the majority (∼85%) of diabetes prevalence (6). These two categories of diabetes have also been correlated with a decreased life expectancy as those living with T1DM and T2DM in the United Kingdom (UK) were estimated to have an average loss in life years of 7.6 and 1.7 years, respectively, compared to the general UK population (7). Classic signs of diabetes include high fasting plasma glucose (above 7 mmol/L), decreased responsiveness to glucose (persistent hyperglycemia after controlled sugar consumption), elevated glycosylated hemoglobin A1c (HbA1c), and the presence of autoimmune markers (beta-cell autoantibodies), with the latter specifically pertaining to T1DM (8). Diagnosing between T1DM and T2DM can be difficult, particularly among adults, as around 5%–15% of patients are diagnosed with T2DM despite having autoantibodies present (9). These findings may suggest that a significant portion of T1DM cases are misdiagnosed as T2DM.

Although the etiology behind T1DM is not fully elucidated, it has been established as a multifactorial disease resulting from the immune-mediated destruction of insulin-secreting pancreatic beta-cells within the Islets of Langerhans. As such, those with T1DM often require frequent exogenous insulin administration to maintain euglycemia. This mainstay treatment of multiple daily insulin injections has the inherent risk of potentially life-threatening hypoglycemia, for those with impaired awareness. On average, individuals with T1DM experience 1 episode of severe, disabling hypoglycemia per year, which can be accompanied by a seizure, coma, or death (10, 11). Preventative measures, such as the continuous glucose monitoring systems, allow those who inject insulin to monitor blood glucose levels more tightly throughout the day. In combination with insulin pumps, the use of a hybrid closed-looped system that continuously monitors blood glucose levels and automatically adjusts the delivery of rapid-acting insulin has been explored and was recently approved by the U.S. Food and Drug Administration (FDA). Although this technology enables those living with T1DM to achieve improved glucose management, continuous accurate insulin infusion may fail from blockages or leakages (12). Furthermore, the glucose monitoring sensors can become less accurate from anomalies including slow sensor signal attenuation, miscalibration, or dislodgment of the sensor from underneath the skin (12). Hence, there remains a struggle to restore normoglycemia and improve glucose management in those living with T1DM without the typical and sometimes life-threatening complications associated with exogenous insulin therapy.

## 2 History of islet cell transplantation

**Figure 1:**
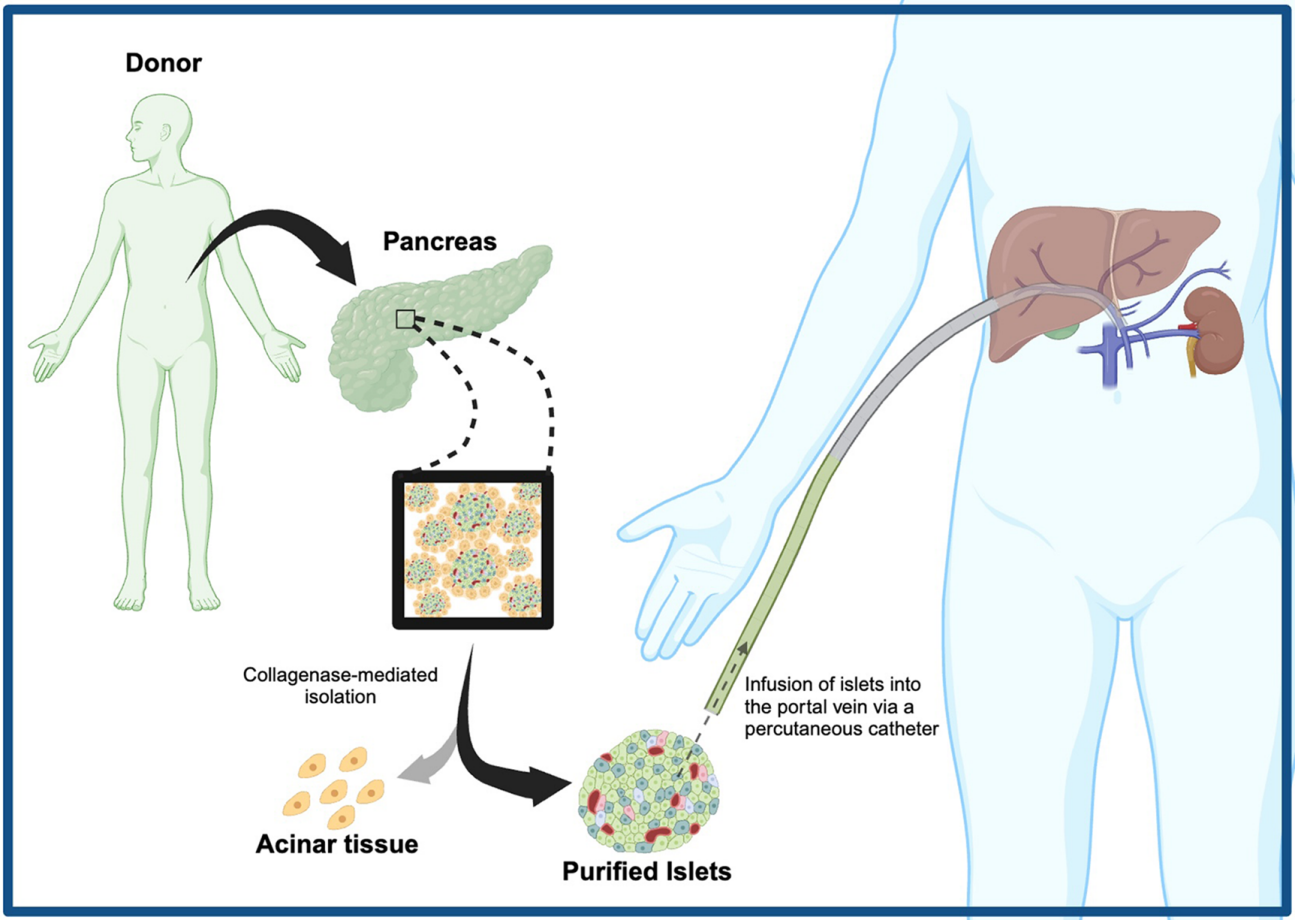
Broad methodology of clinical islet transplantation, involving collagenase-mediated islet isolation, percutaneous infusion of islets into the recipient's portal vein, and engraftment.

Pancreatic islet transplantation (ITx) has become an established approach that frees recipients from severe hypoglycemic events, and insulin injections while improving glycosylated HbA1c. The modality of ITx has been explored as early as 1893, twenty-nine years before the discovery of insulin by Banting and Best. In December of 1893, Watson-Williams and Harsant attempted to treat a 13-year-old boy dying from ketoacidosis by performing the first documented islet tissue transplantation with pieces of sheep pancreas (13). While minor improvement in glycosuria was observed, the boy rejected the xenograft and died comatose 3 days following transplantation with similar outcomes in attempts made in the 50 years thereafter (13, 14). In the 1950s, the hypothesis that the removal of exocrine acinar tissue was paramount in the viability and function of pancreatic grafts was well established (15). With the islets of Langerhans making up a mere ∼2% of the pancreas, islet isolation from adjacent exocrine tissue within the pancreas became a vital step for improving engraftment. This led researchers to perform laborious pancreatic microdissection to remove exocrine tissue under the microscope, resulting in poor yields and quality of islets; consequently, research efforts in the field of pancreatic fragment transplantation declined (16). However, in 1965, Moskalewski introduced the method of collagenase-mediated isolation of guinea pig islets, revamping the field of ITx research (17). In 1972, Ballinger and Lacy demonstrated the first-ever experimental reversal of diabetes in rats through the transplantation of isolated islets within the peritoneal cavity and thigh muscles (18). The following year, Kempt et al. demonstrated that isolated islets infused within the portal vein leading to the liver were the most effective and long-lasting site for the reversal of diabetes in rats, thus establishing a promising clinical site for ITx, one that is still used today (19) (Figure 1). Clinical intraportal ITx first saw success in 1980 where they preserved the endocrine function of 10 patients undergoing pancreatectomies for the treatment of chronic pancreatitis; following collagenase-mediated isolation of patients’ own islets and successful infusion of these islets autografts into the portal vein, they found that four patients achieved insulin independence for at least 1, 9, 15, and 38 months, respectfully (20). Although ITx with autografts would be ideal for decreasing the chances of alloimmune rejection, allograft transplantation (islets received from genetically non-identical donors) has been more frequently explored due to the scarcity of healthy islets in individuals living with T1DM. Despite these thrilling outcomes in ITx, the field failed to see large-scale success as the reproducibility of islet isolation was poor leading to low islet purity and yield. In this period, an automated and standardized islet isolation approach, the Ricordi Automated Method, was not widely employed (21). Consequently, a mere ∼8.2% of the 267 allograft transplant patients treated between 1980 and 1996 achieved insulin independence for greater than one year (22). It was not until 2000 that Shapiro et al. pioneered a breakthrough, the *Edmonton protocol,* reigniting the flame of clinical ITx research (23). The *Edmonton protocol* was the first ITx clinical trial to utilize newer and more potent immunosuppressive agents: sirolimus, tacrolimus and anti-CD25 antibody (daclizumab) (23). The protocol also infused a larger number of healthy islets, isolated with the Ricordi Automated Method, into the portal vein compared to previous clinical studies [11,547 ± 1,604 islet equivalents (IEq) per kg of recipient's body weight] (23). Shapiro et al. performed ITx on seven patients with T1DM, and their glucocorticoid-free (i.e., prednisone-free) immunosuppressive regimen demonstrated effective immunosuppression, circumventing the diabetogenic effects associated with glucocorticoid usage (23). In fact, all seven T1DM patients achieved insulin independence for >1 year with functional insulin secretory function, indicated by sustained circulating C-peptide levels (a peptide co-secreted with insulin) (23). The ground-breaking success of the *Edmonton protocol* sparked worldwide interest, inspiring >1,000 ITx procedures in over 30 International transplant centers in the next two decades (24). However, long-term follow-up of seven T1DM recipients enrolled in the multicenter international *Edmonton Protocol* failed to show sustained islet allograft function over a decade from their first infusion, with only one subject remaining insulin independent (25). The functional mass of islet allografts appears to decrease over time as 20% of recipients remained insulin-independent at 10 years compared to 61% at 1 post-transplant in 255 single-center ITx (26). Where insulin independence may not always be achieved, ITx may still effectively reduce instances of severe hypoglycemic events while promoting euglycemia. A Phase 3 clinical trial by Hering et al. found that infusing islets into the portal vein of forty-eight patients with brittle T1DM led to 87.5% achieving an HbA1c <7.0 with no severe hypoglycemic events at 1-year post-transplant and 71% of patients sustaining these criteria by 2 years (27). More recent studies corroborate these findings as they saw a marked reduction of severe hypoglycemic events up to 10 years post-transplant (28, 29). Despite the marked progress in the field of clinical ITx, the inability of this procedure to sustain long-term insulin independence warrants further developments. Advancements may include further optimizing islet isolation and preparation, exploring alternative transplant sites that offer longevity in islet graft function, testing more effective and less toxic drug regimens, identifying alternative insulin-secreting cell sources (stem cell-derived and xenogeneic), and utilizing novel biomaterials and devices. Through these innovations, cellular replacement therapies may become a more practical, accessible, and promising “functional-cure” for a broader range of people living with diabetes.

## 3 Current status and limitations of islet cell transplantation

### 3.1 Donor selection criteria and islet availability

The limited number of viable islet donors has been a determining factor for the number of people afflicted with T1DM who can undergo ITx. With the human pancreas estimated to hold between 2.3–14.8 million islets (30), isolation outcomes aim to have high purity and yield (>300,000 IEq). Over the past two decades, multiple publications have attempted to correlate donor characteristics to the viability and outcome of islet isolation. Retrospective studies that examined donor body mass index (BMI), age, body weight, tissue cold ischemia time, hospitalization length, and HbA1c found that all these factors correlated with islet isolation outcomes (31–33). Although these investigators strive to identify ideal donor characteristics with these studies, interpretation of results must be done with caution. A large portion of retrospective analysis identifies ideal donor characteristics based on high isolation yields (31–33), but a large number of islets is not always a good indicator of islet function. Herein lies room for error in translating findings to optimal islet physiology and graft performance. This is well illustrated by a study reviewing 153 human islet isolations, in which older donors (age 51–65) produced a significantly higher islet yield (>100,000 IEq) and purity compared to younger donors (age 2.5–18) (34). However, they also measured the insulin stimulation index, an indicator of islet function, and found that younger donor islets demonstrated significantly superior insulin secretory capabilities compared to the group of older donors (34). Therefore, the current method of defining ideal donors in the literature may be misrepresentative of the true definition of an “ideal” donor in terms of optimal islet physiology. Nevertheless, standardizing donor selection for ITx may help to improve long-term success.

The first scoring system based on donor characteristics was developed by O’Gorman et al. and has been used to determine whether a pancreas would be viable for clinical islet isolation (35). The system was developed at a single isolation centre based on 326 donors between 1999 and 2004 (35). More recently, Wang et al. developed the North American Islet Donor Score (NAIDS) to assess pancreas selection for appropriate clinical transplantation with increased accuracy (36). Similar to O’Gorman's system, the NAIDS acts as a diagnostic tool for clinical decision-making based on donor characteristics, however, this system was developed through retrospective multicentre analysis of a larger data set: 1,056 donors across 11 islet isolation centres in North America (36). Moreover, the NAIDS has been validated and remains to be the most useful and available tool for donor pancreas selection to date (37). Certainly, examining outcomes worldwide such as efforts of the Clinical Islet Transplant Registry may refine these scoring systems to assess relationships between primary graft function and donor characteristics (38). More specifically, building criteria based additionally on the relationships between graft performance, length of insulin independence, and islet insulin secretory ability to donor characteristics may be advantageous. Efforts to elucidate this relationship with ITx outcomes may improve the discriminative abilities of the scoring system, allowing clinicians to identify donors that would likely have favourable outcomes in both islet isolation and transplantation.

Akin to other organ transplantation, there is a growing disparity between the availability of donors and the climbing numbers of eligible patients that can benefit from ITx. In 2019, it was estimated that 463 million people worldwide were living with diabetes and its associated complications (39). That same year, the Global Observatory on Donations and Transplantation registered 40, 608 deceased organ donors (40). Assuming that ∼10% of the estimated global diabetes population suffers from T1DM, and that all organ donors were eligible for islet isolation and transplantation, only 0.088% of the global T1DM population could receive a single donor transplant in 2019; patients routinely require multiple islet donors to achieve insulin independence. Furthermore, not all organ donors would fit the eligibility criteria for islet isolation and transplantation. These considerations may suggest that an even lower percentage of those living with T1DM can undergo ITx, widening the disparity between islet supply and treatment demand. Therefore, clinical ITx has been limited to those living with brittle diabetes and life-threatening hypoglycemic unawareness (23). As such, there is a drive to identify a less limited, alternative insulin-producing cell source.

### 3.2 Assessing islet graft function

The significant improvements in experimental ITx research along with clinical advancements demonstrate the ability of researchers to assess allograft or autograft function and adjust protocols accordingly. Having an accurate monitoring system provides investigators with more information on the impacts of treatment. Similar to standardizing ITx protocols and donor selection, creating a scoring system to monitor islets objectively has been a major area of interest. To achieve this, monitoring graft function is a key measure of ITx outcomes, which can help direct new alterations and improvements to current treatment protocols. Furthermore, using multiple indicators to assess graft function can help create standardized and validated scoring systems that eliminate some biases with clinical observation.

Currently, the main factors used as objective measures of graft function are clinical indicators. Measures in controlled glucose tolerance tests, fasted circulating C-peptide, HbA1c, daily exogenous insulin requirements, and renal function have been evaluated individually or in combinations to create standardized scoring systems. To name a few, these systems include the homeostatic model assessment (HOMA) -beta score (41), a secretory unit of islet transplant objects (42), transplant estimated function (43), and most recently the BETA-2 score (44). Visualizing islet grafts may serve as complementary information to the clinical parameters described. Imaging systems evaluated experimentally like positron emission tomography (PET), single-photon emission computed tomography (SPECT), magnetic resonance imaging (MRI), and ultrasound imaging paint a larger picture by visualizing biological processes at a cellular level (45). At present, there is a lack of a standard, clinically relevant, and non-invasive imaging method to monitor islet grafts especially in the intrahepatic site despite the ongoing efforts to validate such an imaging technique.

### 3.3 Limitations of clinical portal vein infusion

As of today, virtually all clinical ITx worldwide are mediated through intrahepatic islet infusion. Although pancreas transplantation can yield similar glycemic outcomes and have comparable costs, ITx is a less invasive procedure that carries lower rates of severe complications (46). While this method of percutaneous intraportal pancreatic islet infusion is a minimally invasive procedure and an effective means to achieve insulin independence, it is not without expected risks including portal vein thrombosis and hypertension, hepatic steatosis, and intraperitoneal bleeding from hepatic punctures (47). Furthermore, despite the refinements made in islet isolation and transplantation protocols over the last two decades, intrahepatic islet transplantation is still associated with an immediate loss of 50%–70% post-transplantation (48, 49). This acute islet cell death in the peri-transplant period compromises long-term treatment success and severely limits engraftment. Furthermore, a larger number of islets is required per recipient, further restricting the number of T1DM patients that can be treated with the already limited donor supply. Factors that may contribute to early graft loss specific to the portal vein microenvironment include the instant blood-mediated inflammatory reaction (IBMIR), activated endogenous liver immune cells, and islet hypoxia. These limitations hold true for both allogeneic and autologous (in cases of chronic pancreatitis) ITx. Addressing these barriers may help identify clinically relevant solutions that could improve early graft survival such as alternative transplant sites.

The instant blood-mediated inflammatory reaction (IBMIR) is a well-studied and early consequence of intrahepatic islet infusion. This complex and nonspecific innate immune response is a major cause of the acute destruction of islets post-transplantation (50). The IBMIR results in a coagulation cascade that is triggered by two islet-specific factors that promote platelet binding: the negatively charged islet surface (51) and the external expression of tissue factor on the islets (52). Since the graft is infused directly into the bloodstream via the portal vein, there is ample opportunity for circulating platelets to interact with these coagulation triggers on the islet surface. Following the formation of macroscopic clots, a panel of cytokines are released, and inflammatory cell recruitment and activated ensue (53). There have been multiple attempts to protect islets against hypoxic and inflammatory stress associated with IBMIR. In experimental models, the coating of islets with endothelial cells (54) and the infusion of anti-coagulates including heparin (55), low-molecular-weight dextran sulfate (56), and thrombin inhibitors (57) have all shown to be effective means of disrupting the IBMIR response. However, to date, only heparin has been validated in clinical settings (58).

Native islets within the pancreas are well-oxygenated, as they make up only 1%–2% of the pancreas’ total volume but receive 10%–15% of its blood flow (59). However, through the isolation process and in culture conditions, they suffer from a drop in oxygen delivery and consequently undergo cell death (60). Unlike full organ transplantation, islet grafts are simply infused into the portal vein and are not anastomosed to blood vessels, thus experience reduced oxygen availability and hypoxia exposure until angiogenesis forms a functional circulatory system within a 10–14-day period following transplantation (61, 62). Hence, islets are mainly oxygenated through diffusion in the early stages of engraftment, which is further impaired by the low oxygen tension of the portal vein system. Moreover, these islets have been shown to experience a persistent and chronic drop in endogenous oxygen tension, going from an initial 40 mmHG within the pancreas to a meager 5 mmHG in the portal vein for up to 3 months post-transplant (59, 63). Consequently, the hypoxic environment is a trigger for cell death, thus resulting in a major loss in islet graft mass. The evident hypoxia among other hepatic factors contributes to poor engraftment, and therefore a larger number of islets is required at this site. In addition, the intrahepatic site still poses a challenge for graft imaging and retrieval, which can be more detrimental in removing malignancies if alternative cell sources are employed. As such, these factors may suggest the liver is not an optimal site for islet transplantation.

### 3.4 Transplant site independent factors contributing to loss in functional islet mass

Though there has been major progress in clinical islet isolation, this extensive 5–7-h multi-step process remains detrimental to functional islet mass. As explored earlier, islet isolation consists of cold enzymatic digestion, which is later followed by mechanical shearing, density gradient purification, and cell culturing. The early development of the Automatic Method utilizing the Ricordi Chamber and continuous digestion-filtration pancreas processing eliminates some human error and has been shown to substantially improve the qualitative and quantitative clinical isolation outcomes (21, 64). Over the next three decades, the Automated Method has evolved to further improve islet isolation outcomes and remains the central technology in clinical islet processing facilities worldwide (15). Despite these advancements, this process can be extremely stressful to sensitive beta-cells, therefore leading to loss in functional islet mass. The current isolation and purification procedures destroy the islet capillary network, thereby preventing the delivery of adequate oxygenation to the level of a normally functioning pancreas. As such, islets experience a period of acute hypoxia throughout isolation, which has been shown to induce apoptosis in beta-cells through upregulating the pro-apoptotic transcription factor C/EBP homologous protein (CHOP) *in vitro* (65). Moreover, evaluation of human islets immediately following isolation revealed that ∼30% of all islets stained positive for apoptosis [terminal dUTP nick end labelling (TUNEL) staining], with beta-cells representing the largest proportion of stained cells (66). Human islets transplanted into immunodeficient nude mice also demonstrated a large loss in functional islet mass as they measured up to a 70% decrease in beta-cell mass by 1-month post-transplant (67). Other murine studies demonstrate a similar trend, with this profound reduction in islet mass being independent of the transplantation site (50, 59).

Another major contributor to graft attrition is the immune response. Recruitment of macrophages following the inflammatory reaction, which is propagated by the IBMIR, results in migration and activation of cytotoxic T cells (CD8+) that directly contribute to islet cell death. These mechanisms will be explored further in the next section; however, it is vital that the recipient's immune reaction is identified as a contributor to the loss of functional graft mass. For this reason, transplant recipients are required to undergo long-term immunosuppression treatment for the length of the graft function in order to prevent rejection. Despite these efforts to prolong graft function, chronic immunosuppression has potentially serious side effects including the risks of developing debilitating infections or malignancies. Furthermore, immunosuppressants used in the past have also been shown to have diabetogenic properties as a result of direct harmful effects on beta-cell function (68–70). Subverting the immune response while minimizing immunosuppressant toxicities remains a major challenge in ITx, though the development of more selective and potent drugs over the years has been majorly beneficial.

## 4 Immunomodulation: a fine line

### 4.1 Overview of the auto- and alloimmune response

The majority of patients undergoing ITx are afflicted with T1DM, and as such, are susceptible to two distinct types of immune-mediated graft destruction: the alloimmune response to the foreign islets, and the recurrent autoimmune response that is the main driver of the initial onset of this metabolic disease. Multiple studies have worked on identifying pathways and immune cells involved, along with determining the roles each contributes to ITx outcomes. Despite the ongoing investigation, there is still an unclear understanding of which process is the main instigator of immune rejection. Nevertheless, distinguishing the mechanisms of these two types of immunity can help generate novel targeted treatments and transplant approaches that could decrease the occurrence of graft rejection.

As many transplant recipients are within the later stages of T1DM, indicated by a large portion of immune cell-mediated beta-cell loss, there is a high probability of recurrent autoimmunity. Before diving into the mechanism of recurrent autoimmunity, it may be useful to further explore T1DM pathogenesis. In 1974, the immune system was first suggested to play a role in T1DM when Nerup et al. discovered an association between the type of human leukocyte antigens (HLA) complex and insulin-dependent diabetes (71). The HLA system, also known as the major histocompatibility complex (MHC), is primarily involved in antigen presentation to elicit a targeted immune response. Specifically, HLA class I (MHC class I) molecules are ubiquitously expressed on the plasma membrane of almost all nucleated cells and present cytosolic peptides to CD8+ T cells through interaction with T cell receptors (TCRs), while HLA class II (MHC class II) is exclusively expressed on B lymphocytes, antigen-presenting cells (APCs), and activated T lymphocytes to detect circulating antigens and present them to CD4+ T cells also via TCRs (72). A heightened sensitivity in MHC results in immune reactions to a larger panel of antigens. As such, the HLA system is responsible for foreign antigen detection, and in cases of autoimmune diseases, endogenous antigens. Since the initial association between HLAs and T1DM, genome-wide association studies corroborate the significance of antigen presentation, as they found that up to 50% of HLA genes (notably HLA class II genes) accounted for the genetic risk of T1DM (73, 74). Furthermore, a study controlling for HLA class II alleles found that HLA class I genes are also associated with T1DM (75). Nonetheless, there remains a lack of consensus on an identified primary autoantigen involved in T1D, possibly due to the heterogeneity of this metabolic disease. Islet-specific autoantigens that have been considered include proinsulin (precursor of insulin), zin transporter 8 protein (essential for biosynthesis and secretion of insulin), insulin promoter factor 1 (IPF-1), islet amyloid polypeptides (peptide hormone co-secreted with insulin), etc (76). There is a general hypothesis that beta-cell autoantigens are processed through the APCs HLA class II molecule complex, resulting in activation of naïve T cells to autoreactive CD4+ T cells. Following activation, these autoreactive T cells migrate to the pancreas and locally release a panel of cytokines to stimulate macrophage and T cell-mediated beta-cell destruction (77). These views are supported by the following findings: (i) the presence of infiltrated T cells in T1DM patient inflamed islets (insulitis) at the onset of T1DM (78); (ii) T cells obtained from within islets of T1DM donors were highly reactive to an autoantigen (preproinsulin) (79); and (iii) systemic immunomodulators targeting T cells delayed disease progression in clinical studies (80). Although the cytotoxic mechanisms driving T1DM remain elusive, there is strong evidence that HLA complex-mediated antigen presentation and T cells are involved in autoimmunity.

Clinical and preclinical experimental outcomes of ITx have been associated with a recurrent autoimmune response. Similar immune events responsible for the onset of T1DM have also been seen in the period following ITx. In non-obese diabetic (NOD) mice, an autoimmune model of T1DM, MHC class II mismatching between donors and recipients demonstrated longer graft survival compared to when the donor and recipient shared similar MHC class II antigens (81). Moreover, studies utilizing a rat model of autoimmune T1DM also found that performing ITx with MHC-mismatched grafts demonstrated prolonged survival and were not susceptible to recurrent autoimmunity compared to MHC-matched grafts (82, 83). Altogether, these preclinical findings provide further evidence of the significant role of MHC antigen presentation in T1DM autoimmunity. In the clinical setting, characterizing recipients’ immune cell reactivity against islet autoantigens pre- and post-transplant can help elucidate the relationship between recurrent autoimmunity and transplant outcomes. A study exploring this relationship performed multivariate analyses of 21 people living with T1DM and demonstrated that the presence of cellular autoimmunity immediately prior to and one year following ITx was associated with significant delays in achieving insulin-independence and inferior graft function, indicated by lower circulating C-peptide levels (84). Furthermore, a study linking clinical ITx to increased rates of post-transplant autoreactive memory T cell proliferation provides greater evidence for recurrent autoimmunity in patients with T1DM (85). Although the mechanisms underlying the recurrent autoimmune response seen in T1DM patients remain unclear, clinical and preclinical findings provide evidence for its occurrence and possible contributions to long-term graft failure.

The alloimmune response is another major concern that contributes to unfavourable ITx outcomes. This process is driven by alloreactive T cells that respond to the genetic dissimilarities between the recipients and the islet graft tissue. Similar to autoimmunity, the MHC complex plays a vital role in eliciting an immune response. Specifically, host immune cells are directed against unfamiliar MHC class I molecules that also ubiquitously present foreign peptides found within the allograft cells, the foreign nature of donor MHC I molecules themselves, and MHC class II on recipient's APCs that uptake circulating foreign antigens originating from the graft (72, 86). CD8+ T cell activation through foreign antigens presentation on allografts, mediated by foreign MHC class I recognition, leads to CD8+ cytotoxic T cell-mediated islet destruction and the triggered release of cytokine that precipitates inflammation and coagulation (86). Consequently, blood flow to the islets becomes disrupted and graft ischemic injury occurs. As mentioned, hypoxic conditions have been linked to cell death and upregulated genes in the cell death pathway which contribute to graft failure and metabolic dysfunction (65, 66). Additional activators of the alloimmune response involve the MHC class II molecules on APCs that present foreign antigens to activate CD4+ helper T cells (72). Subsequently, these CD4+ helper T cells activate macrophage through cytokine release along with promoting the production of antibodies via B lymphocyte activation (86). A study in which investigators reconstituted immunodeficient mice with a mix of lymphocytes that excluded alloreactive T cells demonstrated long-term islet allograft survival using mismatched MHC complex between donor and recipients (87). The alloimmune reaction generated through the mismatch between donor and recipient MHC molecules is a major contributor to allograft rejection.

### 4.2 Overview of immunomodulatory agents used in clinical islet transplantation

The struggle to suppress immune rejection has been an ongoing battle since the first initial islet mass transplant in 1893. Utilizing immunosuppressive agents has allowed the field to achieve and prolong insulin independence; however, there has yet to be any long-term success in curing T1DM. Nevertheless, immunosuppressants have been a major contributor to allogeneic ITx success and have evolved throughout the years. The goal of immunotherapy is for recipients to develop a tolerance phenotype to transplanted donor tissue, thus preserving graft function and survival. In the clinical setting, there are two phases of immunosuppressant treatment to achieve this tolerance: induction and maintenance immunosuppression.

Induction agents target immunity that would be heightened during transplantation to reduce the incidence of acute rejection. Sustained induction therapy would most likely result in iatrogenic events, therefore prolonged use is not ideal. Practically all transplantation employs induction therapy, including ITx, but there is no global standard ITx immune modulation regimen. Typically, induction therapy is employed ∼1–2 days prior to transplantation and is administered up to 7 to 14 days post-ITx. Induction therapy that has T cell-depleting actions has been shown to have a positive effect on long-term insulin independence, regardless of the type of maintenance immunotherapy later employed (88). Thus induction therapy has been and remains a crucial step in clinical ITx (88). The following induction agents are typically used in clinical ITx and the corresponding mechanism of action (Table 1).

**Table 1:** Mechanism and uses of immunosuppressives used in the induction phase of ITx.

Glucocorticoids	Prednisone is a synthetic, anti-inflammatory glucocorticoid that supresses the immune system through altering gene expression. Through binding nuclear receptors, prednisone inhibits the production of proinflammatory cytokines resulting in decreased circulating lymphocytes. Prolonged use of high-dose glucocorticoids has been seen to cause serious adverse effects to systems including musculoskeletal, cardiovascular, gastrointestinal tract, and the endocrine system. Glucocorticoids have also been seen be diabetogenic which further serves as an additional barrier in ITx. Therefore, acute use of these agents in the induction phase of ITx have been explore and are currently still used in the clinical setting (184–186).
Daclizumab (Dac)	Used in the *Edmonton Protocol* (23)*,* Dac is a humanized and monoclonal antibody that inhibits interleukin-2 receptor (IL-2R) via reversible CD25 blockage, the high-affinity subunit on the IL-2R. IL-2 is the main ligand that activates the IL-2R and is released by activated T cells. IL-2 receptors are expressed on a number of immune cells, notably activated T cell, memory CD8+ T cells, naïve T cells, and natural killer T cells (187). Additionally, regulatory T cells (Treg) express IL-2R, and signaling within all these cells promote proliferation, and for some lymphocytes (CD8+ T cells, effector T cells, etc.) are essential for differentiation and activation (187). Dac blockage of IL-2R signaling thus inhibits induction of the immune response, preventing acute immune rejection.
Basiliximab	Targets identical pathways as Dac, but is a chimeric monoclonal antibody produced through recombinant technology (188). Basiliximab blocks the same IL-2R subunit as Dac, and thus supresses immune cell proliferation and maturation. Both Dac and basiliximab are commonly used in renal transplantation to prevent occurrences of acute rejection, and a meta-analysis of 6 randomized controlled trials (total of 509 patients) demonstrated that basiliximab and Dac had similar efficacy and safety profiles (189). Therefore, clinicians have used basiliximab and Dac interchangeably as induction therapy in ITx.
Anti-thymocyte globulin (ATG, Thymoglobulin)	A polyclonal, rabbit anti-thymocyte globulin that rapidly depletes T cells and other lymphocytes. The main mechanism of lymphocyte depletion is complement-dependent cell lysis (190). Consequently, there are less active T cells that can precipitate an allo- and auto-immune reaction in the peri-transplant period. Thymoglobulin has been utilized in a clinical setting for over 30 years (191), and is currently still used as an immunosuppressant in solid-organ transplantation (192).
Tumor necrosis factor (TNF) inhibitor (Etanercept)	Etanercept works through biologically inhibiting pathways involved in the development and progression of inflammation, TNF receptor 1 (TNFR1) and TNF receptor 2 (TNFR2). TNFR1 agonism via the endogenous ligand (TNF-α) binding triggers a proinflammatory response, while activation of TNF2R on immune cells, also by TNF-α, promotes immune cell survival and proliferation (193). Thus, inhibition with etanercept supresses the inflammatory response and immune cell proliferation following transplantation (193). These effects also extend to Treg cells, suppressors of activated immune cells, therefore Etanercept is used with caution in ITx. Moreover, high concentrations of this TNF inhibitor has been revealed to reduce islet function and integrity (194). Etanercept is often used in combination with ATG or alemtuzumab as an ITx induction therapy.
Alemtuzumab	Alemtuzumab is a monoclonal and humanized antibody against CD52 found on the membrane glycoprotein of T and B lymphocytes, NK cells, macrophages, and other immune cells. The function of CD52 is still unclear, however some have suggested that this pathway may be involved in T cell co-stimulation and migration (195) along with Treg induction (196). Therefore, alemtuzumab administration has been seen to cause significant T and B lymphocyte depletion.
Anakinra	Another anti-inflammatory agent typically used with etanercept in the induction phase. Anakinra competitively binds IL-1R, thereby inhibiting the proinflammatory actions of IL-1 (197). Agonism of IL-1R, stimulated via damage recognition (during transplantation), triggers the production of a cascade of inflammatory cytokines including TNF-α (197). Therefore, inhibiting this signaling pathway would be ideal for decreasing the inflammatory and immune recruitment response immediately following ITx.

Following transplantation, higher concentrations of immunosuppressants are initially used to prevent acute rejection and over time, a lower dose is prescribed to decrease the risk of toxicities associated with chronic treatment. This maintenance immunomodulatory regimen aims to protect islet grafts from the allo- and recurrent auto-immune responses that were previously explored. Since this lifelong immunosuppressive therapy is a crucial treatment for transplant recipients, immunosuppressants over the years have constantly evolved to prolong islet function, while still having minimal toxicity. This can be achieved with potent and selective agents with minimal off-target effects. The following agents have been commonly used as maintenance immunosuppressive therapy in ITx, and the associated mechanisms (Table 2).

**Table 2:** Mechanism and uses of immunosuppressives used in the maintenance phase of ITx.

Cyclosporin (Cyclosporine A, CsA)	Early biological studies demonstrated potent immunosuppressive abilities of CsA through blocking the transcription of cytokine genes (IL-2 and IL-4) that are necessary for T cell activation (198, 199). It was later discovered that these effects were mediated through inhibition of the calcium and calmodulin dependent serine/threonine phosphatase, calcineurin. Calcineurin is stimulated via calcium and calmodulin during T cell activation, where it dephosphorylates nuclear factors of activated T cells (NFAT), which activates these proteins and allows them to translocate to the nucleus (200). Once NFAT are in the nucleus, they bind DNA and associate with other transcription factors to promote transcription of cytokines: IL-2, IL-4, IL-10, and IL-17 (201). CsA mediated calcineurin inhibition prevents NFAT dephosphorylation, decreasing the transcription of cytokines that are vital propagators of the allo- and auto-genic immune response. Specifically, this blockage is achieved through CsA binding to the immunophilin, cyclophilin A (predominantly found in T cells) and this complex has enhanced selective affinity for calcineurin, thus inhibiting its phosphatase ability (201). The effect on IL-2 has been thought to be the main contributor of immunosuppression. As explored earlier, IL-2 is necessary for the action, survival, and differentiation of CD4+ and CD8+ T cells (187).
Tacrolimus (FK506, tac)	A macrolide antibiotic that supresses the immune system in a similar way to CsA. However, tac binds to a different immunophilin, the FK506 binding protein (FKBP), which also leads to the inhibition of calcineurin (202). As such, tac and FKBP complex-mediated calcineurin inhibition results in decrease cytokine gene transcription, therefore decreasing T cell proliferation. One main difference between CsA and tac is that the latter is around 100 times more potent, which may be a reason for tac gaining popularity over the years for easier dosage (202). Moreover, tac has the capacity to reverse phases of allograft rejection when the use of steroids becomes ineffective (203). Thus, tac is an ideal agent for maintenance immunosuppression, and was used in combination with sirolimus in the *Edmonton protocol* (23)*.*
Sirolimus (rapamycin)	Veniza and colleagues discovered rapamycin (sirolimus) on Easter Island in the early 1970s and identified it as a product of Streptomyces hygroscopicus (204). Although rapamycin was initially isolated as an antifungal agent, later studies revealed potent immunosuppressive activities through inhibiting the mammalian target of rapamycin (mTOR), a vital component in immune cell maturation, function, and proliferation. mTOR is a serine-threonine kinase that functions through two distinct complexes: mammalian target of rapamycin complex 1 (mTORC1) and mammalian target of rapamycin complex 2 (mTORC2) (205). Rapamycin is proposed to interact with a binding protein [immunophilin FK506-binding protein 1A, 12kDA (FKBP12)] to form a complex that specifically blocks mTORC1 (206). The FKBP12-rapamycin complex binds the amino-terminal of mTORC1, disrupting cell growth by reducing translation, ribosome biogenesis, and autophagy. Moreover, mTORC1 plays a major role in regulating cell growth and downstream processes in immune cell development, thus FKBP12-rapamycin mTORC1 blockade impairs dendritic cell maturation and function, and inhibits T cell and B-cell proliferation (206). In rodents, rapamycin mediated mTOR blockage caused significant thymus atrophy, associated with lower T cell output (207). Moreover, *in vitro* exposure of rodent and human CD4+ CD25+ Treg cells to rapamycin did not impair Treg-dependent immune suppression, and conversely promoted expansion of functional Tregs cells in T1DM patients (208). These potent immunosuppressive effects contribute to the crucial tolerance phenotype necessary in islet engraftment, and is the reason that rapamycin remains one of the most frequently used maintenance immunosuppressive drug in ITx including in the *Edmonton protocol* (23)*.*
Mycophenolate mofetil (MMF)	MMF is a prodrug of mycophenolic acid (MPA) which inhibits *de novo* synthesis of guanosine nucleotides through potent type II inosine monophosphate dehydrogenase (expressed in activated lymphocytes) inhibition (209). This enzyme is a rate-limiting step of the nucleotide synthesis pathway that T and B cells are more dependent on compared to other cell types. Hence, MPA has potent and selective cytostatic effects on lymphocytes (209). MPA has been shown to induce apoptosis in activated T cells, and the guanosine nucleotide depleting effects decrease the expression of selective adhesion molecules required for lymphocyte recruitment and infiltration (209).

### 4.3 Toxicities of immunosuppression

**Figure 2:**
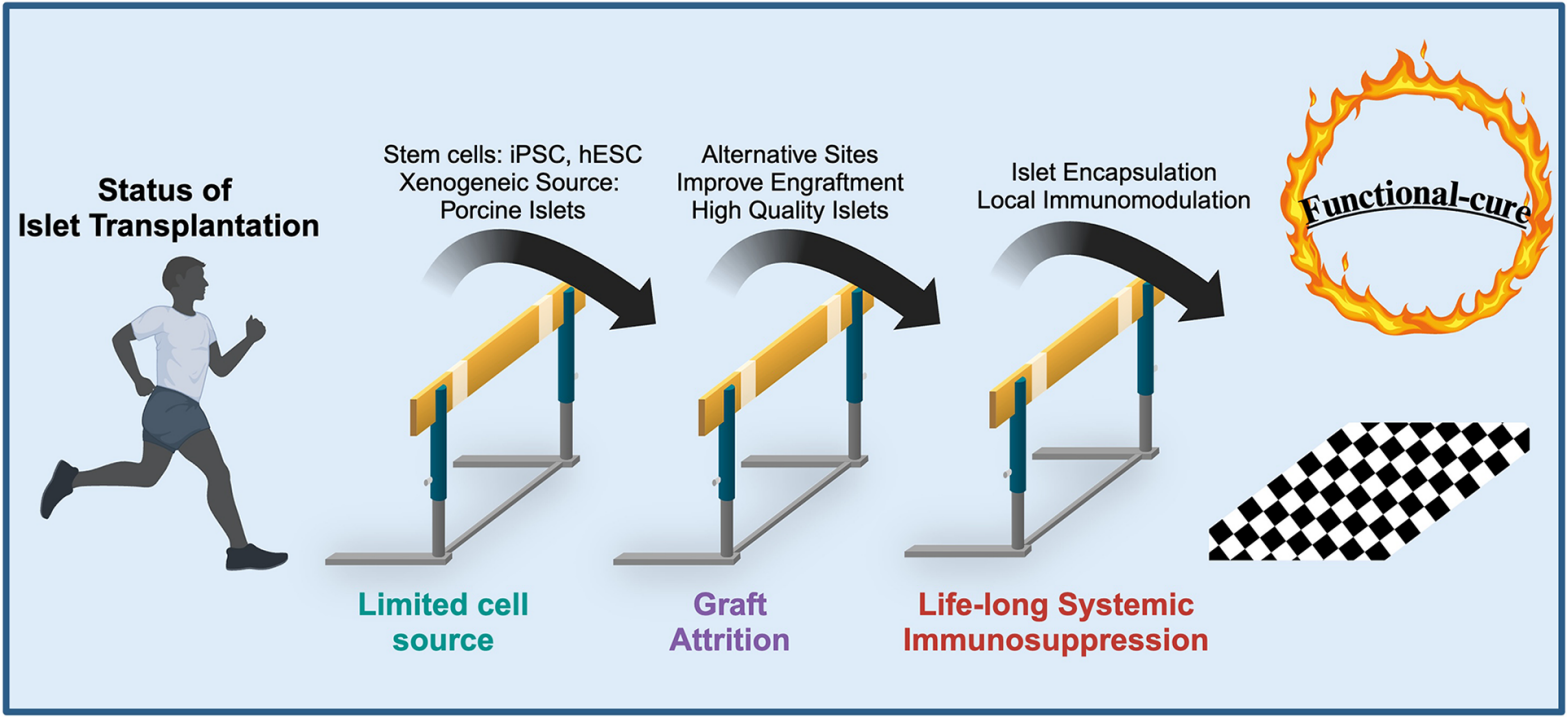
Current hurdles in islet transplantation and solutions that overcome these limitations to provide a “functional-cure” for T1DM. Figure adapted with permission from Desai, 2018 (183).

The marked progress made in the field of transplantation is not without the development of more potent and selective immunosuppressives. Despite ITx consisting of a smaller mass transplanted compared to whole organ transplants, these recipients have one of the most rigorous immunosuppressive regimens (89). As such, multiple toxicities have been associated with the lifelong use of these agents. The chronic immune paralysis in ITx that prevents alloreactivity has been shown to have minor but common risks including mouth ulcers, diarrhea, and acne (90). More life-threatening risks associated are the development of malignancy and serious infection, although these are rare (90). Frequently used immunosuppressives (tacrolimus and sirolimus) have also been shown to have direct toxic effects on beta-cell function and survival, thus being inadvertently diabetogenic (91, 92). These multiple toxicities associated with chronic immune suppression remain a major barrier to improving the quality of life of ITx recipients. In fact, the recipients’ ability to tolerate these toxicities is a factor in the patient inclusion criteria. The need to reduce or even completely abolish the requirement for chronic immunosuppression is one of many major hurdles (Figure 2). Therefore, investigators strive to develop systemic immunosuppressive-free transplant approaches that can be applied to novel and promising extrahepatic transplant sites to effectively subvert the immune response.

## 5 Novel advancements in islet transplantation

### 5.1 Devices and alternative transplant sites

While transplanting islets within the liver currently accounts for virtually all clinical ITx and is an effective means that frees recipients from insulin injections, the procedure often results in acute islet cell death and/or gradual graft attrition due to multiple factors in the intraportal hepatic site (93). Consequently, an estimated ∼70% loss in initial islet mass occurs, meaning that recipients routinely require multiple organ donors to achieve and sustain insulin independence (93). Though effective, ITx into the liver is not the ideal transplant site after considering the hostile nature of the hepatic microenvironment. These considerations previously explored indicate that the liver is not the optimal site for ITx.

To promote engraftment, an ideal ITx site should provide adequate vascularization, substantial space to accommodate for the significant volume of transplanted islets, and sufficient nutrients to aid in islet survival and revascularization. Additionally, avoidance of acute graft loss due to host inflammatory reactions in the peri-transplant period is paramount for decreasing the number of islets required to ameliorate hyperglycemia and can increase the number of T1DM recipients that may be treated. As mentioned, an accessible site would also allow for safety monitoring, non-invasive transplantation, and routine biopsies. Identifying such a site would enable easy retrieval which is ideal for removing abnormal growths associated with alternative cell sources such as insulin-producing stem cells. Considering this all, there are concerted efforts to identify an alternative transplant site.

A multitude of investigators have explored more favourable extrahepatic ITx sites in experimental models and for some in a clinical setting. Experimental sites explored include the liver, spleen, kidney subcapsular space, bone marrow, omentum, peritoneum, intestinal wall, muscle, subcutaneous space, and immune-privileged sites (anterior chamber of the eye) (94). While many of these sites effectively cured hyperglycemia in experimental animal models, translating these successes into a clinical setting remains challenging. For instance, islets allografts infused into the bone marrow of non-diabetic rats effectively produced insulin and had limited rejection (95). Further, ITx in an allogeneic diabetic mouse model demonstrated that bone marrow infusion was superior to the hepatic site in achieving normoglycemia (96). On the contrary, a pilot randomized controlled clinical trial found that all but 1 patients who received an intra-bone marrow islet infusion (*n* = 7) saw a loss in islet graft function within the first 4 months post-transplantation (97). These findings are one of many instances where experimental successes fail to translate to clinical outcomes. Distinct differences in human and animal anatomy, immune system, and physiology may be largely responsible in the divergence of extrahepatic ITx outcomes seen. Coughlan et al. outlines each site for their ability to satisfy the essential characteristics for ITx, and the current status of preclinical and clinical findings (94). Certainly, each site has their respective advantages and limitations in satisfying explored factors (transplant efficacy, ease of monitoring, capacity, oxygen tension, etc.) leading to varying levels of success in preclinical and human trials. Employing biomaterials, novel immune evasion technologies, and angiogenic approaches within these alternative sites may surmount site-specific shortcomings and improve feasibility.

The subcutaneous space is a promising extrahepatic site for ITx due to its minimal invasiveness, ability to support a large transplant volume or device, and potential for monitoring transplant function (98–100). Despite these benefits, the subcutaneous site is a poorly vascularized location, contributing to a hypoxic environment and thus islet cell apoptosis. The majority of vascularized connective and supportive tissue surrounding islets are lost during islet isolation (15); therefore delivering islets in devices have been explored to recapitulate the endogenous pancreas, promoting engraftment and survival in the subcutaneous space (101). To achieve such a feat, Barkai's group designed a bioartificial pancreas (*Beta-Air* from *Beta-O2 Technologies Ltd.*) that suspends islets in an alginate hydrogel with sufficient oxygen (via a refillable gas chamber and gas permeable membrane), an external barrier providing immune protection, and a mechanically protective frame (101). Their subcutaneous implanted device was able to reverse diabetes for up to 6 months in streptozotocin-induced diabetic rats using allogeneic islets (101). These exciting results led to a clinical trial where 4 diabetic patients underwent subcutaneous implantation with 1–2 of their bioartificial pancreas, however, outcomes were not as triumphant (102). All four patients saw no changes in metabolic control and low levels of circulating C-peptide, but transplanted islets within the device survived up to 3–6 months post-implantation (102). However, the recovered bioartificial pancreases demonstrated insufficient *ex vivo* function, as there were low glucose-stimulated insulin responses (102). Another undesirable outcome seen in these patients was the signs of a substantial foreign-body reaction, indicated by immune cell accumulation in the surrounding areas of implantation (102). This lifelong complex and dynamic process involves continuous protein adsorption, immune and proinflammatory cell recruitment, and extracellular remodeling which can all contribute to the failures associated with subcutaneously transplanted biomaterials (103). Hence, alternative strategies that do not utilize permanently implanted devices (a trigger of the foreign-body response) whilst still promoting early vascularization have been heavily explored. This necessary step can involve preconditioning the subcutaneous site with implanting biomaterials such as angiogenic growth factor-loaded polylactide capsules (104), methacrylic acid copolymer coated biomaterial (105), or vascular access catheter (106) that are subsequently removed prior to islet transplantation. Pepper et al. developed a “device-less” approach that harvests the foreign-body response, demonstrating that pre-implanting and then later removing catheters (at 4-weeks post-implant) sufficiently vascularizes the subcutaneous ITx site (106). Subsequent syngeneic ITx into this preconditioned site effectively reverses diabetes in mice (>100 days) with a marginal number of islets, while diabetic mice that underwent subcutaneous transplantation without any preconditioning failed to achieve normoglycemia (106). This promising and novel “device-less” transplant modality is currently being explored as of 2021, in a phase I clinical trial consisting of 5 patients with T1DM (ClinicalTrial.gov Identifier NCT05073302). Another method that also promotes early angiogenic growth, but does not require preconditioning the subcutaneous site, was designed by Nalbach et al. where they fused islets to microvascular fragments (107). This combination was seen to highly enhance *in vitro* angiogenic activity and effectively restore normoglycemia with a subtherapeutic number of microvascular-fused islets transplanted within the dorsal skin fold of diabetic mice (107). A similar subcutaneous approach that delivered a bioabsorbable methacrylic acid bounded polymer with islets supported graft revascularization and survival (108). Though, the application of bioabsorbable materials may also support prevascularization as Kuppan et al. effectively vascularized a subcutaneous site with a nanofibrous polymer scaffold functionalized with angiogenic factors, promoting the survival and function of porcine islets that were later transplanted in mice (109). Although these strategies show promise, there has yet to be an established alternative islet transplant site used in the clinical setting. Moreover, patients would still be subject to chronic immunosuppressive toxicities regardless of transplant location. Further experimental and clinical progress in novel subcutaneous transplant modalities may one day lead to such an extrahepatic ITx site that can overcome the barriers explored.

### 5.2 Alternative cell sources

#### 5.2.1 Xenogeneic islets

The use of islets that originate from different species has been widely explored in order to circumvent the demand for human donors. Multiple sources of xenogeneic islets have been investigated including tissue derived from bovine (110), fish (111), sheep (112) and porcine (pig) (113). Although each source has associated advantages and disadvantages, at present, pig islets prove to be the most promising source due to their similar physiological and morphological features to human islets. Additionally, pigs are an attractive source because of their (i) high fecundity, (ii) efficiency of genetic modification through well-established techniques, (iii) practicality of housing them under pathogen-free conditions, and (iv) cost-efficient feasibility (114). Furthermore, porcine insulin has been used as an established and effective diabetes therapy for over 2 decades, demonstrating that pig islets may serve as a promising alternative cell source for ITx (115). Multiple preclinical studies support this notion; xenotransplantation of neonatal porcine islets has been demonstrated to be an effective means of achieving long-term reversal of diabetes in diabetic rodents (116) and nonhuman primate (NHP) models (117, 118) in adjunct to immunosuppressant treatment. However, translating these experimental successes to a clinical setting remains a major challenge. The first ever human islet xenotransplantation of 10 patients with fetal porcine islets was performed by Growth et al. in 1994 (119). They demonstrated that xenografts can survive for up to serval months, but patients failed to show any improvements in their glycemic control (119). Since then, investigators have made procedural adjustments including genetic modifications, islet encapsulation, and isolation modifications in an attempt to improve the clinical success of xenotransplantation. In 2014, Matsumoto et al. transplanted 14 patients with unstable T1DM with encapsulated neonatal porcine islets without immunosuppression and saw a reduction in unaware hypoglycemic events, but only a minimal reduction in HbA1c and daily insulin dosages at 1-year post-transplant (120). A similar 2016 clinical study in Argentina, where 8 patients underwent intraperitoneal transplantation with encapsulated neonatal porcine islets, also saw patients experiencing fewer episodes of unaware hypoglycemia and an improvement in HbA1c, but no change in daily insulin injections (121). Beyond ITx, recent thrilling advancements in clinical xenotransplantation highlighted the genetic modification approach. Two separate patients were transplanted with a genetically modified pig heart or kidney with 10 and 69 gene edits, respectively (122, 123). Triumphantly, no acute rejection was seen with both procedures with the pig heart failing at 48 days and the pig kidney at just under two months after transplantation. These thrilling cases shed light on the possibility of a functional alternative organ source that may help fill the demand for organ transplantation. Exploring the clinical transplantation of islets from these genetically modified pigs may address the immunological barriers of xenotransplantation. As islet isolation disturbs the intricate vasculature perfusing islets, significantly lower exposure to xenogeneic antigens on donor endothelial cells would be expected in comparison to whole-organ transplants, where the whole-organ vasculature is derived from porcine. Despite the acute hypoxic conditions preceding angiogenesis as described, this process may ensure reduced exposure to additional donor tissue and reduce the risk of rejection. While the two pig-to-human heart and kidney transplant mechanism of rejection is still under investigation, we propose reducing exposure to xenogeneic endothelial cells may lead to more sustainable outcomes in xenogeneic ITx of these modified porcine strains. Moreover, the overall lower transplant mass in tandem with these genetic modifications may support more sustained long-term islet graft function. Examining their utility in clinical studies would serve as a confirmation. Certainly, more work is necessary in the field to provide long-term function, and if successful, could represent an unlimited source of organs.

#### 5.2.2 Stem cells

Another alternative cell source that has been heavily investigated for ITx is stem cells. The attractive benefits of stem cell therapy include the unlimited cell source and their suitability for immune tolerance. There has been increasing interest in functionalizing and generating insulin-secreting cells from human embryonic stem cells (hESCs) and induced pluripotent stem cells (iPSCs). To achieve such a feat, extensive steps are required to differentiate these stem cells into glucose-responsive and insulin-producing cells. In 2001, Assady et al. demonstrated that hESCs could spontaneously differentiate into an array of cell types, including those that produce insulin (124). Following this discovery, Segev et al. modified a differentiation protocol initially used to generate insulin-producing cells from mouse ESCs and were able to successfully differentiate hESCs to secrete a substantial amount of insulin (125). Further exploration of hESC differentiation protocols led to the successful production of more mature, glucose-responsive, and insulin-expressing endocrine cells (126). In 2008, this protocol was used to generate hESC-derived pancreatic endoderm that was transplanted into diabetic-induced mice, effectively reversing hyperglycemia and indicating the potential for clinical usage (127). The alternative stem cell source, iPSC, is a promising approach due to the possibility of an autologous transplantation. The protocol to dedifferentiate human skin fibroblast to human pluripotential stem cells was first discovered by Yamanaka's group in 2006 (128). Following dedifferentiation mediated through the Yamanaka genetic factors (Oct3/4, Sox2, c-Myc, and Kl4) (128), many demonstrate the ability of iPSCs to differentiate into insulin-producing beta-cells (129, 130). Even so, their ability to form mature pancreatic endocrine cells remains inferior to products from hESC protocols (131), proving an area for further developments in iPSC differentiation protocols. Potential for iPSC is apparent as autologous stem-cell derived islet infusion into the portal vein of a T2DM patient effectively reduced their exogenous insulin requirement, all without immunosuppression (132). Furthermore, ITx of allogeneic stem cell-derived islets restored insulin independence in two T1DM patients and ongoing transplants are being conducted (133). Although intense lifelong suppression may not be required with autologous iPSC-based transplantation as there is an absence of allorecognition, the possibility of recurrent autoimmunity is an area of concern. Furthermore, hESC and iPSC differentiation strategies require major monetary and time investments, which may bring into question their feasibility. Nevertheless, recent advancements in stem cell-derived islet approaches (134) have led to promising clinical trials. ViaCyte developed a pancreatic endoderm cell (S4) product derived from hESC, known as PEC-01, which matured into glucose response insulin-producing cells after several months *in vivo* (127–136). These encouraging findings led to the first in human clinical trials in 2014 (NCT02239354), which encapsulated PEC-01 within a cell-impermeable membrane to circumvent immunosuppression (137). While endocrine cells were observed post-explantation, device fibrotic overgrowth resulted in graft loss and no evidence of insulin secretion, ultimately leading to the trial being terminated (138). In 2017, subsequent follow-up trials (NCT03163511) utilized a porous membrane to allow for direct graft vascularization, but this necessitated systemic immunosuppression. Results published one-year post-transplant, demonstrated glucose-responsive c-peptide secretion, confirming the *in vivo* differentiation of the pancreatic progenitors. Histological analysis of explanted grafts, demonstrated that while most of the cells stained positive for the neuroendocrine marker chromogranin A, these endocrine cells co-stained mostly for glucagon and few for insulin. In addition, host fibroblasts were abundant indicative of a robust foreign body reaction (138–140). Critically, these studies demonstrated no serious safety concerns, such as teratoma formation, but emphasized the importance of mitigating foreign body reactions.

The field began to further optimize methodologies and protocols to terminally differentiate stem cell-derived islets (>S6) to become insulin-secreting in a glucose-responsive manner *in vitro*, prior to transplantation (141, 142). These refinements led to cell products that demonstrated improved glucose control *in vivo* when transplanted into diabetic rodents. Subsequently, in 2021, Vertex Pharmaceuticals initiated a series of clinical trials (NCT04786262) with a more mature cell product (VX-880), infused into the portal vein under immunosuppression to avoid fibrosis. Interim-published abstracts have demonstrated improved patient glycemic control and insulin independence (133), albeit delayed in comparison to rodent students for reasons that have yet to be fully elucidated. These pioneering clinical trials highlight the auspicious potential stem cell-derived islets possess for the management of T1D. However, they also underscore the unmet challenges that persist, preventing this beta cell replacement therapy from becoming a standard of care. These include but are not limited to, (1) optimizing differentiation protocols to increase maturity, at a functional and transcript level, (2) eliminating off-target cell types, (3) developing strategies (e.g., genetic engineered, biomaterials) to circumvent the necessity of anti-rejection drugs, (4) identify the ideal transplant environment which is highly vascularized while possessing muted stress responses and (5) refine large-scale manufacturing processes. Regardless, PSCs may one day soon serve as an alternative cell source for ITx.

### 5.3 Biomaterial strategies - localized immune modulation

Localized immune modulation is an attractive alternative and a potential replacement for systemic immunosuppression. Targeting immune and inflammatory cells exclusively at the transplant site could help overcome off-target debilitating toxicities associated with chronic systemic immunosuppression. In this framework, researchers utilize two main approaches (i) islet encapsulation to prevent contact with immune cells, and (ii) localized and sustained release of immunomodulatory agents (143). If these methods prove effective, there is a high possibility that the requirement for extensive systemic immunosuppression will be abolished (Tables 1, 2). Adapting natural and/or synthetic biomaterials has been rigorously investigated.

#### 5.3.1 Islet encapsulation

The current strategies that utilize biomaterial-based islet encapsulation include macroencapsulation, microencapsulation, and nanoencapsulation, which are characterized based on islet-to-host distance (144). These approaches all work as a physical barrier that protects transplanted islets from immune cell attack, while still enabling them to identify changes in blood glucose levels and subsequently secrete insulin into the circulation. Moreover, these biomaterials must allow for sufficient diffusion of nutrients, oxygen, and metabolic waste, promoting islet survival. The macroencapsulation approach houses the largest number of islets within devices and has the largest islet-to-host distance. Thus, a main disadvantage of this approach is the limited diffusion of oxygen and nutrients which can be detrimental to graft viability and function (145). To overcome these drawbacks, devices such as the bioartificial pancreas (Beta-Air) employ a gas chamber that provides exogenous oxygen to the islets (101). Apart from the experimental success that was previously discussed, where diabetic rats achieved long-term normoglycemia with allogeneic islets implanted with Beta-Air devices (101), immunosuppressive-free xenotransplantation in diabetic NHP utilizing this marcoencapsulation technology also demonstrated sustained graft function for up to 6 months (146). The requirement for tedious oxygen refills within the gas chamber represents a major drawback to such an approach. An innovative self-sustaining approach by Wang et al. may address this limitation as they recycle a cellular waste product, carbon dioxide, to generate oxygen via a chemical reaction (147). Their device, termed inverse breathing encapsulation device (iBED), showed promise in a proof-of-concept mice and minipig studies whereby islet graft function was sustained up to 3 months. Alternative oxygen-generating approaches employing chemical reactions and electrophoresis have been explored (148–150). Another macroencapsulation approach that overcomes some challenges with diffusion is intravascular devices. These are hollow semi-permeable fiber devices that house islets within the lumen and directly connects them to host arteries (145). The semipermeable membrane effectively protects islets from immune-mediated damage, but the blood-device interaction can bring rise to blood coagulation (145). To avoid excess thrombosis, Song et al. designed an intravascular device using silicon nanopore membranes and demonstrated superior *in vivo* hemocompatibility, pore size selectivity, and hydraulic permeability to typical devices that employ polymer membranes (151).

The microencapsulation approach involves encapsulation of a single or small cluster of islets within microcapsules. These strategies minimize the islet-host distance and their often spherical configuration allows for greater diffusion (larger surface area to total volume ratio) relative to macroencapsulation devices (145). Conformal coating has been an ideal approach, but the risk of incomplete shielding and islet antigens breaching the capsule barrier leaves grafts susceptible to immune attack. The most used microencapsulation materials that are capable of forming spherical capsules around islets are hydrogels, a crosslinked three-dimensional network that can be derived from a wide array of natural and synthetic polymers (144, 145). Extensive polymer modifications promote crosslinking that improves viscoelasticity, hydrophilicity, and shape retention within an aqueous environment of the body, thereby making hydrogels a biocompatible material that can mirror endogenous tissue (152). Furthermore, their semi-permeable membrane permits oxygen, nutrients, and waste exchange from encapsulated islets, while still protecting against immune infiltration and activation. In terms of islet microencapsulation, researchers have investigated a variety of natural and synthetic derived hydrogels: alginate (153), agarose (154), collagen (155), polyvinyl alcohol (PVA) (156), poly(lactic-co-glycolic acid) (PLGA) (157), etc. These hydrogel varieties show promise in animal models of diabetes, effectively conferring immune protection and prolonging the viability and functionality of transplanted allogeneic islets (153, 154, 156, 157). The experimental success of microencapsulation-mediated immune cloaking of islets has given rise to clinical trials exploring immunosuppressive-free alternative cell approaches. Although no clinical trials transplanting microencapsulated xenogeneic or allogeneic islets have demonstrated excellent long-term metabolic control (158), a modest reduction in exogenous insulin usage and hypoglycemic episodes was seen in patients transplanted with microencapsulated porcine islets without immunosuppression (121).

The thinnest barrier, and thus the closest distance between the islets-to-host, is the nanoencapsulation approach. This strategy encapsulates each individual islet with a nano thin coating. By significantly minimizing the coating thickness, diffusional distance also decreases and hence improves islet responsiveness to glucose fluctuations and increases oxygen, nutrients, and insulin diffusion (145). Furthermore, permeability can be simply modified by controlling coating thickness and composition, in comparison to the other encapsulation approaches that require larger alterations, i.e., altering membrane size, number of islets encapsulated, or encapsulation material (159). There are two main methods for islet nanoencapsulation: “PEGylation” and layer-by-layer (LBL) assembly. The former method involves a cell surface modification with poly(ethylene glycol) (PEG), a synthetic polymer that can be modified with chemical groups (acrylates), enabling the formation of crosslinked bioinert networks around islets (160). This technique, termed “PEGylation”, covalently attaches PEG to islet surfaces with aims to improve biocompatibility via enhancing hydrophilicity, decreasing direct protein adhesions (preventing complement and coagulation cascade), and cloaking islets from immune attack. However, complete immune-blocking effects were not seen in the diabetic NHP model as transplantation of PEGylated allogeneic islets failed to restore euglycemia, even in conjunction with immunosuppressives (161). The alternative nanoencapsulation approach, LBL assembly, has shown more promise. As the name implies, LBL assembly involves the deposition of alternating nano thin films on an islet surface. The ease of altering the biomaterials and the number of deposited layers used provides researchers the ability to tune and optimize the nanoencapsulated structures. For example, Zhi et al. demonstrated that 8 chitosan/alginate bilayers provided superior *in vivo* immune protection of allogeneic islets compared to 4 bilayers in mice, revealing a relationship between structure thickness and immune isolation abilities (162). In terms of biomaterials used, researchers have explored the incorporation of immunomodulatory materials in these bilayers to locally subvert the immune response. Dr. Hubbert M. Tse's group in Alabama generated an LBL assembly multilayer coating with tannic acid, a natural immunomodulatory polyphenol, and poly(N-vinylpyrrolidone) (PVPON), a biocompatible non-toxic polymer (163). The formation of hydrogen bonds between these two distinct layers increases structural stability and coating retention around islets. Further, in NOD mice, the tannic acid/PVPON nanoencapsulation approach exhibited reduced *in vivo* immune cell infiltration, pro-inflammatory chemokine synthesis, and significantly delayed allo- and auto-immune rejection compared to nonencapsulated islets (163). These thrilling findings may spearhead further developments, possibly inspiring the use of more potent and specific immunomodulatory agents that target negative regulators pathways of T cell immune function [etc., cytotoxic T-lymphocyte-associated antigen 4 (CTLA-4), programmed death 1 (PD-1)]. Further, the adaptability of the LDL assembly approach can underpin a multilayer nanocoating with more than 2 distinct layers, further enhancing localized immunosuppression.

#### 5.3.2 Alternative methods for localized immune modulation

These strategies enable clinicians to fine-tune local drug release, which they can adapt based on the period of engraftment. ITx recipients typically require a larger dose of anti-inflammatory or immunosuppressives in the peri-transplant period, later tapering off to lower doses for long-term usage. To accommodate these fluctuating dose requirements, exploring the use of a temperature-dependent elastin-like peptide, developed by Kojima and Irie, to locally deliver drugs at the site of ITx may serve as a promising solution (164). This technology could allow for greater control in local drug administration through locally changing temperatures at the transplant site (given its feasibility) which would trigger drug release. Devices that house islets and contain a refillable drug reservoir can also provide the opportunity for a controlled localized drug release. The Neovascular Implantable Cell Homing and Encapsulation (NICHE) device, developed by Paez-Mayorga et al., precisely fulfills such a role and effectively restored euglycemia in a T1DM rat model (165). Another alternative method that can support the transplant site microenvironment is co-transplanting islets with cells that secrete immunomodulatory cytokines (166). Cells explored for this approach include mesenchymal stem cells (167), Tregs (168), Sertoli cells (169) and dendritic cells (167). However, there are limiting factors to this approach including the source of these immunomodulatory cells and their requirement for long-term survival and function.

#### 5.3.3 Drug-eluting biomaterials

The local and sustained release of immunosuppressives and anti-inflammatories is a strategy that can enhance engraftment, promote tolerance, and improve ITx success by creating less hostile microenvironments. Rather than functionalizing a barrier that confers protection from immune cell contact, this approach typically permits cell infiltration which aids engraftment and islet vascularization. Thus, locally targeting immune infiltrating cells is desirable and can be achieved with synthetic scaffolds, nanoparticles, and microparticles along with cell-based strategies (170, 171). Furthermore, in comparison to systemic immunosuppression, this approach that confines drug release at the site of transplant most likely can achieve a higher local concentration with less off-target toxicities. If this notion holds true, local drug-releasing technology may have a larger therapeutic window that can enable clinicians to amplify desirable immunosuppressive and anti-inflammatory effects.

To achieve localized immunomodulation, synthetic materials have been heavily explored due to material homogeneity and ease of structure fabrication and modification, contributing to a reliable and controlled system (172). The following are commonly used FDA-approved synthetic materials utilized experimentally for drug delivery in ITx: poly(lactic-co-glycolic acid) (PLGA), polylactide co-glycolide (PLG), poly(ethylene glycol) (PEG), and poly(vinyl alcohol) (PVA) (170, 171, 173). These biodegradable materials gradually deteriorate within the host system, releasing a sustained mass of agents that were incorporated within these structures. The most explored forms of this emerging ITx approach include layered scaffolds that entrap drugs, or spherical micro- and nan-particles that encapsulate chemical agents within. Liu and colleagues explored the scaffold delivery approach via implanting streptozotocin-induced diabetic mice with an allogeneic islet-containing multilayered microporous PLG scaffold impregnated with transforming growth factor-beta 1 (TGF-ß1), an immunomodulatory cytokine (174). *in vitro* drug kinetics of TGF-ß1, found within layers of the scaffold, demonstrated a burst initial release in the first 3 days (>90% total mass) (174). Implantation of this scaffold within the epididymal fat pad decreased inflammatory cytokine (TNF-α, IL-2, monocyte chemotactic protein-1) production by at least 40%, corresponding to a 60% drop in leukocyte infiltration and significant delay in allograft rejection compared to empty scaffolds (174). Another group, utilizing a polydimethylsiloxane scaffold to deliver fingolimod (FTY720, Gilenya), an immunosuppressive that inhibits effector T cell recruitment and migration (175), demonstrated a similar *in vitro* burst release with the bulk of the drug being released in the first 7 days (176). However, no significant improvements were seen when this scaffold was implanted in the epididymal fat pad of diabetic mice (176). The length of drug release could be an explanation for varying levels of experimental success with scaffold drug delivery. As discussed, in clinical ITx, recipients are required to endure lifelong immune suppression to prevent allo- and auto-immune graft rejection. Within this framework, a burst initial drug release may not be effective at subverting the immune response long-term, thereby failing to achieve prolonged graft function. Hence, researchers have explored alternative methods for achieving a longer sustained local drug release.

Synthetic drug-eluting particles are a promising technology to achieve sustained local immunomodulation. As mentioned, PLGA is a biodegradable and FDA-approved synthetic material that has typically been employed for controlled drug delivery (173). Drug-eluting micelles fabricated from PLGA or other polymers have also been utilized in other therapeutic applications including chemotherapy, ocular and neurological drug delivery, and vaccines (177). As such, the popularity of applying this promising technology to ITx has increased over the years. In terms of maintenance immunosuppression in ITx, a longer sustained release is ideal; researchers have explored polymer-based microparticles (MP) as they typically elute drugs slower and for a longer duration than their smaller counterpart, nanoparticles (178). Recently, Kuppan et al. developed such a system where they encapsulated dexamethasone (dex), a systemically diabetogenic and anti-inflammatory glucocorticoid, in PLGA MP (179). Their formulation eluted dex *in vitro* for at least 30 days and co-localizing these MPs with allogeneic islets transplanted under the kidney capsule of diabetic mice resulted in a two-fold increase of recipients that achieved euglycemia for 60 days post-transplantation compared to empty MP controls (both groups received a short course of CTLA-4-Ig injections to block of T-cell costimulation) (179). Furthermore, they also saw significantly reduced proinflammatory cytokine expression and increased Treg localization within the grafts of the dex-MP treated recipients (179). Other groups have explored the use of sustained immunosuppressive drug-eluting MPs in the context of experimental ITx. Pathak's group fabricated PLGA encapsulated tacrolimus (FK506), a commonly used maintenance immunosuppressive in clinical ITx (Table 2), and co-delivered this biotechnology with xenogeneic islets within the subcutaneous space of streptozotocin-induced diabetic mice (180). By 30 days post-transplant, the mice that received these FK506-eluting MP were euglycemic (60% survival), while mice transplanted with islets alone all became hyperglycemic by day 15 (180). An alternative MP system, formulated by Fan et al. in Singapore, draws on polycaprolactone (PCL) and PLGA polymers to fabricate two distinct types of rapa-eluting MPs that release drugs at different rates (181). Combining these two particles allowed them to have an initial burst release, via porous PCL MPs, followed by a sustained PLGA MP-mediated rapa release for up to 30 days *in vitro* (181)*.* Rapa is another maintenance immunosuppressive that is widely used in clinical ITx (Tables 1, 2), and co-transplanting this drug-eluting MPs system with allogeneic islets within the anterior chamber of the eye demonstrated a modest (10 day) delay in graft rejection compared to MP containing no drugs (181). Conversely, nanoparticles’ tendency to burst release may be ideal for certain tolerance induction strategies (178). Bryant and colleagues demonstrate this clinically attractive approach by utilizing PLG nanoparticles to deliver donor antigens intravenously, inducing long-term donor-specific tolerance for allogeneic islets transplanted in diabetic mice (182). Despite these limited results highlighting the potential of nano- and micro-particles in subverting the immune response, further investigation into these approaches in the context of ITx is necessary. Optimizing this technology may one day lead to a replacement of chronic systemic immunosuppression where ITx recipients would only require occasional MP administrations.

## 6 Discussion

To date, T1DM still afflicts many individuals worldwide and has been established as an autoimmune-driven disease. Although the mainstay treatment of exogenous insulin injections can help T1DM patients achieve normoglycemia, the daunting risk of life-threatening hypoglycemia unawareness remains interconnected. Islet transplantation has been established as an effective means of reducing these events and granting recipients a period freed from insulin injections; limitations in the field include poor cell survival in the hepatic site, limited donor supply, toxicities of chronic systemic immunosuppression, and long-term deterioration of graft function. Thus, the procedure is typically restricted to those who suffer from brittle diabetes. Nevertheless, the status of clinical ITx today is not without the ground-breaking progress made in the field ever since the first islet tissue transplant in 1893. Further advancements in validating more favourable extrahepatic transplant sites, alternative cell sources, and biomaterial-based localized immunomodulation can help surmount the barriers explored. Innovations within these domains may underpin an ITx approach that requires a lower dose of islets with a sustained long-term function, all while reducing the requirement for lifelong immunosuppression and associated toxicities. If successful, ITx may become a treatment option for a wider range of individuals living with diabetes. Concerted efforts are ongoing to make this a reality with the hopes of translating these preclinical successes into favourable patient outcomes.
